# Coronary microvascular dysfunction and obstructive coronary artery disease in sepsis-associated myocardial injury: authors’ reply

**DOI:** 10.1186/s13054-026-06422-x

**Published:** 2026-11-02

**Authors:** Samantha Lörstad, Per Åstrand, Patrik Gille-Johnson, Yunzhang Wang, Christina Ekenbäck, Fadi Jokhaji, Felix Böhm, Patrik Hjalmarsson, Shajan Shekarestan, Tomas Jernberg, Sara Tehrani, Kambiz Shahgaldi, Jonas Persson

**Affiliations:** 1 Department of Clinical Sciences, Division of Internal Medicine, Karolinska Institutet, Danderyd University Hospital, Stockholm, Sweden; 2 Internal Medicine Clinic, Danderyd University Hospital, Stockholm, Sweden; 3 Infectious Diseases Clinic, Danderyd University Hospital, Stockholm, Sweden; 4 Department of Clinical Sciences, Karolinska Institutet, Danderyd University Hospital, Stockholm, Sweden; 5 Department of Clinical Sciences, Division of Cardiovascular Medicine, Karolinska Institutet, Danderyd University Hospital, Stockholm, Sweden

To the Editor,

We thank Zhou and Hua [1] and Wei and Wu [2] for their interest in our study and their valuable comments [3]. As both letters address overlapping themes, we respond to them jointly, focusing on the thresholds used to define coronary microvascular dysfunction (CMD), CMD as a potential manifestation of systemic microvascular dysfunction in sepsis, and the implications of our angiographic findings for selective coronary evaluation.

## Thresholds for coronary microvascular dysfunction

The thresholds used to define CMD, index of microcirculatory resistance (IMR) > 25 and microvascular resistance reserve (MRR) ≤ 3, were derived from studies of chronic coronary syndromes and ischaemia with non-obstructive coronary arteries, rather than studies in sepsis [4, 5]. Nevertheless, they provide useful reference limits. In structurally normal hearts, the upper reference limit for IMR is approximately 25 [6], suggesting that values above this threshold reflect abnormal coronary microvascular physiology. In sepsis, potential mechanisms include altered vasomotor regulation (endothelial injury, reduced adenosine responsiveness and increased resting flow), extravascular compression due to myocardial oedema and elevated filling pressures, and microvascular obstruction, alongside pre-existing CMD. Their relative contributions remain unknown.

In the absence of sepsis-specific thresholds, these standardised criteria enabled comparison with a matched cohort of patients with chronic coronary syndromes [3]. At the time of IMR and MRR assessment, all patients were haemodynamically stable and vasopressor-free (*n* = 49; mean arterial pressure 89 ± 13 mmHg; heart rate 82 ± 14 beats/min) [3]. Future studies should establish sepsis-specific reference values for coronary microvascular vasodilatory capacity and hyperaemic resistance (e.g., MRR and IMR) and evaluate their prognostic value for clinically relevant outcomes, including mortality, incident heart failure and organ dysfunction.

## CMD as part of a broader cardiovascular phenotype

We agree with Wei and Wu that the association of CMD with impaired ventriculo-arterial coupling, right ventricular–pulmonary arterial uncoupling and right ventricular systolic dysfunction, but not with conventional left ventricular indices, suggests that CMD may represent a coronary manifestation of systemic microvascular dysfunction in sepsis. Potential mechanisms include systemic inflammation, endothelial and glycocalyx injury, and microvascular thrombosis [7]. The thin-walled right ventricle, normally perfused throughout the cardiac cycle, may be particularly vulnerable to altered perfusion pressure when pulmonary vascular resistance and right ventricular afterload increase [8]. However, these cross-sectional associations may be confounded by illness severity and do not establish causality. Microvascular physiology was assessed in the left anterior descending artery (LAD), the standard vessel for invasive coronary microvascular assessment. We acknowledge that single-vessel measurements may not capture regional heterogeneity, particularly in the right coronary territory supplying the right ventricle.

## From protocol angiography to selective coronary evaluation

Zhou and Hua correctly note that protocol coronary angiography, performed for mechanistic purposes in patients selected on the basis of sepsis and high-sensitivity cardiac troponin T (hs-cTnT) ≥ 15 ng/L irrespective of symptoms, does not establish which patients clinicians should refer for coronary investigation. The proportion of patients with obstructive coronary artery disease (CAD) we observed (12/55, 22%) applies to stabilised patients able to undergo invasive assessment, not to all patients with sepsis and elevated troponin. We agree that deciding whom to assess for obstructive CAD and determining the management of identified obstructive CAD are distinct clinical decisions. The recently published Fifth Universal Definition of Myocardial Infarction is consistent with this approach [9]. It emphasises that acute illness, including sepsis, may precipitate atherothrombosis (primary myocardial infarction), while leaving the decision to perform angiography to clinical judgement. Under this definition, secondary myocardial infarction (formerly type 2) is confirmed by obstructive CAD without acute coronary pathology or by a new ischaemic regional wall-motion abnormality.

As requested, Table 1 compares patients without obstructive CAD with those with obstructive CAD, stratified by percutaneous coronary intervention (PCI) during the index hospitalisation. Cardiovascular risk factors clustered among patients with obstructive CAD, whereas ischaemic ECG changes did not discriminate between groups. The highest recorded hs-cTnT values overlapped widely between groups and were lowest in patients who underwent PCI.

**Table 1:** Pre-angiography characteristics by angiographic group

	Obstructive CAD, index PCI (*n* = 6)	Obstructive CAD, no PCI (*n* = 6)	No obstructive CAD (*n* = 43)
≥ 2 conventional risk factors^a^, n (%)	5 (83)	5 (83)	19 (44)
Ischaemic ECG changes, n (%)	3 (50)	3 (50)	17 (40)
Regional wall-motion abnormality^b^, n (%)	1 (17)	4 (67)	7/40 (18)^b^
hs-cTnT, ng/L, median (IQR)	83 (50–159)	225 (217–407)	98 (59–220)

^a^Hypertension, diabetes mellitus, current or former smoking

^b^Regional wall motion could not be assessed in three patients because of inadequate image quality

Six patients underwent PCI during the index hospitalisation, three involving the left main coronary artery (two extending into the LAD or left circumflex artery [LCx]) and three involving a single non-left-main vessel (LAD, *n* = 2; LCx, *n* = 1). Among the six patients with obstructive CAD who did not undergo PCI during the index hospitalisation, two had chronic total occlusions, two had small-vessel disease (< 2.5 mm), one had proximal right coronary artery stenosis managed with clinical follow-up, and one declined intervention. One patient without obstructive CAD at the index angiography underwent repeat angiography and PCI two months later for unstable angina. All patients with obstructive CAD received guideline-directed medical therapy and cardiovascular follow-up. Thus, coronary angiography informed clinical management in all 12 patients with obstructive CAD during the index hospitalisation.

A post hoc exploratory receiver operating characteristic (ROC) analysis was conducted using variables available before coronary angiography (Supplementary Tables S1–S4 and Supplementary Figure S1). The number of conventional cardiovascular risk factors (hypertension, diabetes mellitus, and current or former smoking) showed the greatest discriminatory ability for obstructive CAD (area under the curve [AUC] 0.69, 95% CI 0.55–0.83), followed by hypertension (0.66, 95% CI 0.55–0.77), regional wall-motion abnormality (RWMA; 0.62, 95% CI 0.46–0.78) and highest recorded hs-cTnT (0.62, 95% CI 0.42–0.82). A model combining hypertension, diabetes mellitus, RWMA and hs-cTnT had an apparent AUC of 0.73 (95% CI 0.54–0.91), decreasing to 0.64 (95% CI 0.45–0.82) after bootstrap optimism correction, the model without hs-cTnT (0.64, 95% CI 0.47–0.81).

Given the small sample size and lack of external validation, these findings are insufficient to support a clinical decision rule. Nevertheless, these findings suggest that conventional cardiovascular risk factors and RWMA may help inform selective coronary evaluation, whereas the incremental value of hs-cTnT appears limited. This is consistent with Wei and Wu’s emphasis on risk factors and suggestive clinical features.

Larger prospective studies are needed to develop and evaluate diagnostic algorithms for identifying obstructive CAD and to determine whether algorithm-guided interventions improve clinical outcomes in this setting.

## Supplementary Information

Below is the link to the electronic supplementary material.

Supplementary Material 1: Additional file 1: Supplementary Tables S1–S4 and Supplementary Figure S1

## Data Availability

The datasets used and/or analysed in this correspondence are available from the corresponding author on reasonable request.

## Abbreviations

AUC: Area under the curve
CAD: Coronary artery disease
CMD: Coronary microvascular dysfunction
ECG: Electrocardiogram
hs-cTnT: High–sensitivity cardiac troponin T
IMR: Index of microcirculatory resistance
IQR: Interquartile range
LAD: Left anterior descending artery
LCx: Left circumflex artery
MRR: Microvascular resistance reserve
PCI: Percutaneous coronary intervention
ROC: Receiver operating characteristic
RWMA: Regional wall–motion abnormality
