# Supplementary material for: Coronary microvascular dysfunction and obstructive coronary artery disease in sepsis-associated myocardial injury: authors’ reply
Source: Crit Care. 2026 Nov 2;30:560. doi: 10.1186/s13054-026-06422-x (PMC13726314; doi:10.1186/s13054-026-06422-x)
Supplement: Supplementary file 1 [file 13054_2026_6422_MOESM1_ESM.docx]

# Supplementary Material: authors' reply

## Supplementary methods

This exploratory post hoc analysis included all 55 patients in the COMTESS study who underwent coronary angiography, of whom 12 had obstructive coronary artery disease (CAD) (1). Candidate predictors were limited to variables available before angiography: conventional cardiovascular risk factors (hypertension, diabetes mellitus, current or former smoking, and their number, 0–3), ischaemic changes on the admission ECG, regional wall-motion abnormality (RWMA) on echocardiography and highest registered high-sensitivity cardiac troponin T (hs-cTnT). RWMA could not be assessed in three patients because of image quality.

Discrimination for obstructive CAD was assessed with receiver operating characteristic (ROC) curves and the area under the curve (AUC), with 95% confidence intervals by the DeLong method. Because of the small number of events, a single combination of bedside variables (hypertension, diabetes mellitus and RWMA) was examined, with and without highest registered hs-cTnT (log-transformed), using Firth-penalised logistic regression in patients with complete data (n = 52). Internal validation was performed by bootstrap resampling (1,000 resamples). In each resample the Firth model was refitted and its AUC was estimated in both the resample and the original data; the mean difference (optimism) was subtracted from the apparent AUC. Approximate 95% CIs for the optimism-corrected AUC were obtained by shifting the DeLong confidence limits of the apparent AUC by the estimated optimism. Analyses were performed in R (packages pROC and logistf). Given 12 events, estimates are hypothesis-generating, and no decision rule is proposed.

## Supplementary Table S1. Clinical risk factors and obstructive CAD

| **Variable** | **Obstructive CAD (n = 12)** | **No obstructive CAD (n = 43)** | **AUC (95% CI)** |
| --- | --- | --- | --- |
| Hypertension, n (%) | 11 (92) | 26 (60) | 0.66 (0.55–0.77) |
| Diabetes mellitus, n (%) | 5 (42) | 11 (26) | 0.58 (0.42–0.74) |
| Current or former smoking, n (%) | 9 (75) | 25 (58) | 0.58 (0.44–0.73) |
| Number of risk factors (0–3), median (IQR) | 2 (2–2) | 1 (1–2) | 0.69 (0.55–0.83) |

**Abbreviations:** AUC, area under the receiver operating characteristic curve; CAD, coronary artery disease; CI, confidence interval; IQR, interquartile range.

## Supplementary Table S2. ECG and biomarker findings and obstructive CAD

| **Variable** | **Obstructive CAD (n = 12)** | **No obstructive CAD (n = 43)** | **AUC (95% CI)** |
| --- | --- | --- | --- |
| Ischaemic changes on admission ECG, n (%) | 6 (50) | 17 (40) | 0.55 (0.39–0.72) |
| hs-cTnT, ng/L, median (IQR) | 202 (88–288) | 98 (59–220) | 0.62 (0.42–0.82) |

**Abbreviations:** AUC, area under the receiver operating characteristic curve; CAD, coronary artery disease; CI, confidence interval; hs-cTnT, highest recorded high-sensitivity cardiac troponin T; IQR, interquartile range.

## Supplementary Table S3. Echocardiographic findings and obstructive CAD

| **Variable** | **Obstructive CAD (n = 12)** | **No obstructive CAD (n = 40)*** | **AUC (95% CI)** |
| --- | --- | --- | --- |
| RWMA, n (%) | 5 (42) | 7 (18) | 0.62 (0.46–0.78) |

* Regional wall-motion not assessable in three patients because of image quality.

**Abbreviations:** AUC, area under the receiver operating characteristic curve; CAD, coronary artery disease; CI, confidence interval; RWMA, regional wall-motion abnormality.

## Supplementary Table S4. Combined bedside models for obstructive CAD

| **Model** | **Apparent AUC (95% CI)** | **Optimism-corrected AUC (95% CI)** |
| --- | --- | --- |
| Hypertension + diabetes mellitus + RWMA | 0.70 (0.54–0.87) | 0.64 (0.47–0.81) |
| Hypertension + diabetes mellitus + RWMA + hs-cTnT | 0.73 (0.54–0.91) | 0.64 (0.45–0.82) |

Firth-penalised logistic regression in patients with complete data (n = 52; 12 with obstructive CAD); highest recorded hs-cTnT log-transformed. Optimism correction by 1,000 bootstrap resamples with the model refitted in each resample; 95% CIs for corrected AUCs obtained by shifting the apparent DeLong limits by the estimated optimism. **Abbreviations:** AUC, area under the receiver operating characteristic curve; CAD, coronary artery disease; CI, confidence interval; hs-cTnT, high-sensitivity cardiac troponin T; RWMA, regional wall-motion abnormality.

**Supplementary Figure S1.** **ROC curves for obstructive CAD**. (A) Individual predictors (n = 55; RWMA, n = 52; 12 with obstructive CAD). (B) Combined bedside model, with and without highest recorded hs-cTnT (n = 52; 12 with obstructive CAD); apparent and optimism-corrected AUCs shown. Abbreviations: AUC, area under the curve; CAD, coronary artery disease; CI, confidence interval; DM, diabetes mellitus; HT, hypertension; hs-cTnT, high-sensitivity cardiac troponin T; ROC, receiver operating characteristic; RWMA, regional wall-motion abnormality.

**Reference**

1. Lörstad S, Åstrand P, Gille-Johnson P, Wang Y, Ekenbäck C, Jokhaji F, et al. Coronary microvascular function in patients with sepsis and myocardial injury: an invasive coronary physiology study. Crit Care. 2026;30:(1):351. https://doi.org/10.1186/s13054-026-06179-3
